# Insights into ecological role of a new deltaproteobacterial order *Candidatus* Acidulodesulfobacterales by metagenomics and metatranscriptomics

**DOI:** 10.1038/s41396-019-0415-y

**Published:** 2019-04-08

**Authors:** Sha Tan, Jun Liu, Yun Fang, Brian P. Hedlund, Zheng-Han Lian, Li-Ying Huang, Jin-Tian Li, Li-Nan Huang, Wen-Jun Li, Hong-Chen Jiang, Hai-Liang Dong, Wen-Sheng Shu

**Affiliations:** 1 State Key Laboratory of Biocontrol, Guangdong Key Laboratory of Plant Resources, School of Life Sciences, Sun Yat-Sen University, 510275 Guangzhou, China; 2 Department of Geology and Environmental Earth Science, Miami University, Oxford, OH 45056 USA; 3 State Key Laboratory of Biogeology and Environmental Geology, China University of Geosciences, 430074 Wuhan, China; 4 School of Life Sciences, University of Nevada Las Vegas, Las Vegas, NV 89154 USA; 5 Nevada Institute of Personalized Medicine, University of Nevada Las Vegas, Las Vegas, NV 89154 USA; 6 Guangdong Magigene Biotechnology Co. Ltd., 510000 Guangzhou, China; 7 School of Life Sciences, South China Normal University, 510631 Guangzhou, China; 8 State Key Laboratory of Biogeology and Environmental Geology, China University of Geosciences, 100083 Beijing, China

**Keywords:** Microbial ecology, Biodiversity, Biogeochemistry, Microbial ecology, Biogeochemistry

## Abstract

Several abundant but yet uncultivated bacterial groups exist in extreme iron- and sulfur-rich environments, and the physiology, biodiversity, and ecological roles of these bacteria remain a mystery. Here we retrieved four metagenome-assembled genomes (MAGs) from an artificial acid mine drainage (AMD) system, and propose they belong to a new deltaproteobacterial order, *Candidatus* Acidulodesulfobacterales. The distribution pattern of *Ca*. Acidulodesulfobacterales in AMDs across Southeast China correlated strongly with ferrous iron. Reconstructed metabolic pathways and gene expression profiles showed that they were likely facultatively anaerobic autotrophs capable of nitrogen fixation. In addition to dissimilatory sulfate reduction, encoded by *dsrAB*, *dsrD*, *dsrL*, and *dsrEFH* genes, these microorganisms might also oxidize sulfide, depending on oxygen concentration and/or oxidation reduction potential. Several genes with homology to those involved in iron metabolism were also identified, suggesting their potential role in iron cycling. In addition, the expression of abundant resistance genes revealed the mechanisms of adaptation and response to the extreme environmental stresses endured by these organisms in the AMD environment. These findings shed light on the distribution, diversity, and potential ecological role of the new order *Ca*. Acidulodesulfobacterales in nature.

## Introduction

Iron and sulfur are biologically important elements that are cycled dynamically between the geosphere and biosphere. Microorganisms capable of catalyzing dissimilatory redox transformations of sulfur and iron had a profound impact on Earth evolution [1, 2], and thus have drawn extensive attention from different research fields. Both elements participate in the formation of many minerals, particularly pyrite, which is ubiquitous in nature [3]. When pyrite and other sulfide minerals are exposed to air and water at Earth’s surface, microbially catalyzed oxidative dissolution occurs, generating acid mine drainage (AMD) [4]. AMD, typically characterized by extreme acidity and elevated concentrations of metals and sulfate, represents an extreme habitat to life, as well as a major global environmental challenge [5]. Iron and sulfur oxidations are the primary biochemical transformations occurring in AMD [6], and hence numerous studies have revealed microbial diversity, metabolic functions, and ecological roles of iron- and/or sulfur-oxidizing microbes affiliated with *Acidithiobacillus*, *Leptospirillum*, “*Ferrovum*”, *Ferroplasma*, *Thermoplasmatales*, and the ARMAN (archaeal Richmond Mine acidophilic nanoorganisms) in AMD ecosystems [7–10]. Moreover, the acidophiles involved in the subsequent reduction of oxidized sulfur and iron species (sulfate and ferric iron reduction) in AMD ecosystems cannot be neglected because they are thought to retard AMD generation and contribute to AMD bioremediation [11–13]. So far, a few studies have surveyed the taxonomic diversity of acidophilic sulfate-reducing microorganisms (aSRMs) in AMD ecosystems [12, 14, 15], and several pure cultures of aSRMs have been studied, such as *Desulfosporosinus acidiphilus*, *Desulfosporosinus acididurans*, and *Desulfovibrio* sp. TomC [16–18]. Even so, some abundant microorganisms in AMD ecosystems remain elusive.

**Fig. 1:**
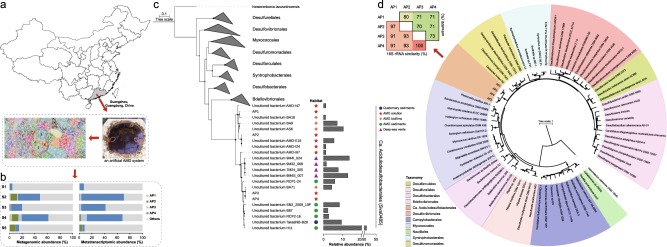
The reconstructed genomes of *Ca*. Acidulodesulfobacterales. **a** Geographical location of an artificial AMD system from which AMD samples were collected, with ESOM image highlighting the four genomes within metagenomic data. **b** Relative metagenomic abundance and metatranscriptomic abundance of the four *Ca*. Acidulodesulfobacterales genomes. S1 through S5 represent July 2016, August 2016, December 2016, February 2017, and August 2017 samples, respectively. **c** A maximum-likelihood phylogenetic tree of 16S rRNA genes from four genomic bins and representatives of the class *Deltaproteobacteria*, and the abundance distribution pattern of *Ca*. Acidulodesulfobacterales in natural environments. **d** Phylogenetic placement of the four bins and their orthoANI and 16S rRNA similarity values. The maximum-likelihood phylogenetic tree was constructed using the concatenated alignment of 16 ribosomal proteins. Bootstrap values were based on 100 replicates, and percentages ≥50% are shown with black circles

In the 2000s, uncultured bacteria BA71 and BA18, affiliated with the deltaproteobacterial Candidate Sva0485 clade were first reported and speculated as potential sulfate/iron reducers, accounting for 4.30 and 1.08%, respectively, of the 16S ribosomal RNA (rRNA) gene clone library of a slime biofilm from the Richmond Mine [19]. Since then, numerous studies have affirmed the presence of the Sva0485 clade (relative abundance ≥ 5%) in AMD ecosystems (including water, sediments, and biofilms) [20–24], including Xiang Mountain, China (Fig. 1c), where they make up 51.1% of a 16S rRNA gene clone library in an AMD sediment [25]. This clade was also found in diverse environments, such as Fe nodules from Quaternary sediments (relative abundance, 9.4%), an inactive hydrothermal sulfide chimney in the deep sea (relative abundances, 12.3–13.8%), and a deep ferruginous sediment from Lake Towuti (relative abundances, ~2–20%) [26–28]. The clade is considered to be composed of potential sulfate-reducing bacteria (SRB) as it is commonly found in active sulfate-reducing consortia [29–31]. However, because of the lack of microbial isolates or genomes, the physiology and ecological role of this taxon remains unknown.

In this study, four draft genomes belonging to the Sva0485 clade were retrieved from AMD metagenomes and the name “*Candidatus* Acidulodesulfobacterales” (*Ca*. Acidulodesulfobacterales) is proposed for this taxon. Then, we surveyed the distribution patterns of these species in AMDs across Southeast China and one artificial AMD system, and evaluated the deterministic factors explaining the spatial distribution pattern of *Ca*. Acidulodesulfobacterales. In addition, the potential roles of these new species in nature were probed by reconstructing their metabolic pathways and examining in situ gene expression by metatranscriptomics. This study expands our understanding of the biogeography, taxonomic diversity, and ecological role of this new order *Ca*. Acidulodesulfobacterales.

## Materials and methods

### Sampling, physicochemical analyses, DNA and RNA extraction and sequencing

An artificial system for simulating and tracking the oxidation of natural pyrite was established with material collected from Yunfu Pyrite Mine, Guangdong Province, on 26 June 2009 in a ventilated greenhouse on the Sun Yat-sen University campus (Fig. 1a) [32]. The details of this artificial system and the pyrite oxidation experiment were described in our previous study [32]. In July 2016 (S1), August 2016 (S2), December 2016 (S3), February 2017 (S4), and August 2017 (S5), representing a seasonal cycle of summer–winter–summer, approximately 50 L of acidic water was pre-filtered using 3.0 and 0.8 μm filters (149 mm diameter; Whatman) to remove coarse particles and eukaryotes [33], and then the filtrate was concentrated to ~500 mL by tangential flow filtration system (PES membrane, 1000 kDa pore size; Merck Millipore, Germany). Each concentrate was divided equally into two parts: one for DNA extraction and the other for RNA extraction, and then was centrifuged (10,000 × *g*, 10 min, 4 °C) to obtain a cell pellet. The samples for RNA extraction were preserved in 1 mL of RNAlater (Ambion) and all samples were stored at −80 °C until nucleic acid extraction. Physicochemical characteristics were measured as previously reported [34], and summarized in Supplementary Table S1. The detailed methods are provided in the Supplementary Information, including details of genomic DNA and RNA isolation and sequencing.

### Genome-resolved metagenomic analysis

Of raw reads generated by Illumina HiSeq and MiSeq sequencers, duplicates were removed as artifacts using an in-house perl script, and unique reads were filtered to remove low quality bases/reads using Sickle (version 1.33) with the parameters “-q 20 -l 50” [35]. After that, all high-quality datasets were co-assembled using SPAdes (version 3.11.0) with the parameters “-k 21, 33, 55, 77, 99, 127 --meta” [36]. To calculate scaffold coverage, all high-quality reads from metagenomic datasets were mapped to the assembled scaffolds (length ≥ 2000 bp) using BBMap with the parameters “minid = 0.97, local = t”. These scaffolds were binned using MetaBAT (version 0.32.4) with the parameters “-m 2000 --unbinned” [37], which considers both tetranucleotide frequencies and the coverage of these scaffolds. The retrieved bins from MetaBAT were evaluated for taxonomic assignment, genome completeness, potential contamination, and strain heterogeneity, using CheckM [38], and were visualized using ESOM [39]. Afterwards, a cluster of four bins belonging to the Sva0485 group was further optimized to obtain high-quality genomes as previously described [8].

### Analyses of genome bins

Four high-quality genomes were submitted to the JGI IMG/MER system for gene calling and annotation [40]. Subsequently, predicted gene functions were manually curated and revised by comparisons with the databases including NCBI-nr, KEGG, and eggNOG. Based on gene annotation, metabolic pathways were constructed for these bins. The 16S rRNA gene sequences were identified using RNAmmer [41] and were then used to search for the closely related 16S rRNA gene sequences in NCBI GenBank using BLASTn. Hits with an alignment coverage ≥ 85% and sequence identity ≥ 85% were downloaded for subsequent analysis. Calculation of relative abundance and transcript abundance was described in the Supplementary Information.

### Phylogenetic analyses

Datasets consisting of 16 ribosomal proteins [42] from the four bins, along with 65 genomes belonging to the *Delta-* and *Epsilon*-*proteobacteria* and one genome from the phylum *Euryarchaeota* were individually aligned using MUSCLE (version 3.8.31) [43], and then were trimmed to remove columns composed of ≥ 95% gaps and the taxa with < 50% of the expected alignment columns using TrimAL with the parameters (-gt 0.95 -cons 50) [44]. The curated alignments were concatenated for phylogenetic analyses, and a maximum-likelihood tree was constructed using RAxML (version 8.1.24) [45], with the parameters set as “-f a -n boot -m PROTGAMMALG -c 4 -e 0.001 -# 100”. In addition, a total of 79 16S rRNA gene sequences belonging to the *Deltaproteobacteria* were aligned using MUSCLE (version 3.8.31) [43], and then the alignment was filtered through TrimAL to remove columns comprised of ≥ 95% gaps, generating a final alignment containing 79 taxa and 1861 alignment positions. The 16S rRNA gene tree was constructed using RAxML (version 8.1.24) [45], with the parameters set as “-f a -m GTRGAMMAI -n boot -c 4 -e 0.001 -# 100”. In addition, combined with the reference DsrAB data set from a previous study [46], a total of 506 DsrAB sequences were used for tree construction using RAxML with the parameters set as “-f a -m PROTGAMMAIJTT -n boot -c 4 -e 0.001 -# 100”. The newick files with the best tree topology were uploaded to iTOL [47] for visualization and formatting.

### Data collection for meta-analysis

To reveal broader patterns in the distribution of *Ca*. Acidulodesulfobacterales in the AMD environment, we collected microbial data and site properties of 59 AMD samples across Southeast China [34] for meta-analysis. A total of 131,720 quality reads were downloaded from the European Nucleotide Archive database (accession no. PRJEB9908) [48]. These reads were combined with the V4 region of 16S rRNA gene sequences from *Ca*. Acidulodesulfobacterales and then were clustered into operational taxonomic units (OTUs) at the 97% similarity level with the UPARSE pipeline [49]. Relative abundance of each OTU was calculated as previously reported [50].

### Statistical analyses

All statistical analyses were implemented using SPSS 18.0, SigmaPlot 10.0, and various R packages (http://www.r-project.org). The relationships between relative abundances/transcript abundances of the retrieved bins and physiochemical properties were assessed using the Spearman’s Rho/Pearson correlation. Redundancy analysis was carried out to identify environmental parameters that could explain the variation in relative abundance of the OTUs related to *Ca*. Acidulodesulfobacterales. Multiple linear regression (MLR) with stepwise method were conducted to test the significance between the relative abundance of *Ca*. Acidulodesulfobacterales and environmental properties. To quantify the contributions of the selected environmental variables and geographical distance to the *Ca*. Acidulodesulfobacterales abundance, we used the Lindeman–Merenda–Gold method in the relaimpo package [51]. In addition, orthologous average nucleotide identity (orthoANI) [52] was calculated among genomes from *Ca*. Acidulodesulfobacterales.

### Accession numbers

The genomes reported in this study are available at the JGI IMG/MER under the Study ID Gs0128962 (accessions: Ga0325886–Ga0325889) and also at the NCBI GenBank under the BioProjectID PRJNA517999 (accessions: SGBB00000000–SGBD00000000 and SHMQ00000000).

## Results and discussion

### A new order *Ca*. Acidulodesulfobacterales in the class *Deltaproteobacteria*

In this study, a total of approximately 434 GB metagenomic raw data were generated for five samples (Supplementary Table S2). The Sva0485 clade was abundant (4.7–61.5%) in all samples (Fig. 1b) and comprised four distinct metagenomic bins, designated AP1-4. AP1-4 formed a distinct clade within the *Deltaproteobacteria* in a concatenated ribosomal protein tree (Fig. 1d). Therefore, we propose that AP1-4 represent a new order and designate it as *Candidatus* Acidulodesulfobacterales (*Ca*. Acidulodesulfobacterales). The 16S rRNA gene sequence identities and OrthoANI values between four bins were estimated, ranging from 91 to 100 and 70 to 80%, respectively (Fig. 1d). 16S rRNA genes have been widely used for taxonomic assignment and phylogenetic relationship determination, as the “gold standard” in both microbial phylogeny and ecology studies for several decades [53–55]. However, some counter examples have been reported that different species shared ≥ 97% of 16S rRNA sequence similarity, such as *Bacillus psychrophilus* and *Bacillus globisporus* (99.8%), and *Serpula hyodysenteriae* B78 and *Serpula innocens* B256 (99.5%) [55, 56]. This phenomenon was also observed in our research, where AP1 and AP2 and AP3 and AP4 shared 97 and 100% 16S rRNA sequence similarity, despite relatively low ANI values of 80 and 73%. As their OrthoANI values were much lower than proposed species cutoff values of 95% [57], they were considered to be four new species affiliated with two new genera, *Candidatus* Acididesulfobacter (*Ca*. Acididesulfobacter, including AP1 and AP2) and *Candidatus* Acidulodesulfobacterium (*Ca*. Acidulodesulfobacterium, including AP3 and AP4) of *Ca*. Acidulodesulfobacterales. The details of their taxonomic epithets are provided in the Supplementary Information.

### The biogeography of *Ca*. Acidulodesulfobacterales in AMD environments

**Fig. 2:**
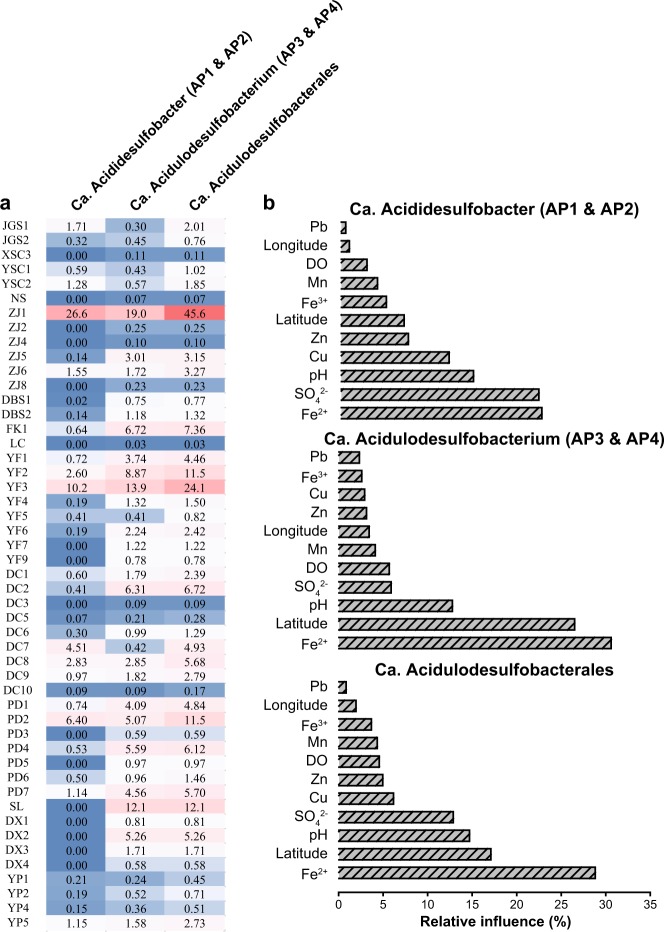
Environmental distribution of *Ca*. Acidulodesulfobacterales. **a** The relative abundance of *Ca*. Acidulodesulfobacterales in AMDs across Southeast China. **b** Relative influence (%) of environmental properties and geographical distance on the relative abundance of *Ca*. Acidulodesulfobacterales

To reveal the spatial distribution pattern of *Ca*. Acidulodesulfobacterales in AMD environments, we reanalyzed microbial data in 59 AMD samples across Southeast China generated by 454 pyrosequencing [34]. As shown in Fig. 2a, members of *Ca*. Acidulodesulfobacterales were nearly ubiquitous, being present in 49 samples, with large differences in their total relative abundances from 0.03 to 45.4%. The genera *Ca*. Acididesulfobacter (AP1 and AP2) and *Ca*. Acidulodesulfobacterium (AP3 and AP4) were similarly widespread and abundant. Subsequently, MLR analysis with multivariate models indicated that ferrous iron was a major factor correlating with the abundance of *Ca*. Acididesulfobacter, *Ca*. Acidulodesulfobacterium, and their order *Ca*. Acidulodesulfobacterales, accounting for 22.6, 30.6 and 28.8% of the relative influence, respectively, followed by latitude, sulfate, and pH (Fig. 2b). For *Ca*. Acididesulfobacter, Cu and Zn were also important factors.

### General genomic features of *Ca*. Acidulodesulfobacterales and their dynamics in an artificial AMD system

The basic genomic characteristics of the four *Ca*. Acidulodesulfobacterales genomes are summarized in Table 1. Their genome sizes were between 1.9 and 2.5 Mb with estimated completeness between 92 and 94% and <1% contamination. In comparison with *Ca*. Acidulodesulfobacterium (AP3 and AP4), *Ca*. Acididesulfobacter (AP1 and AP2) had larger genomes and lower GC contents. The number of predicted genes ranged from 1805 to 2208, and the gene annotation rate ranged from 79 to 86, 79 to 86, and 66 to 78% in the NCBI-nr, KEGG, and eggNOG databases, respectively. Interestingly, the AP1 genome had more CRISPR loci and higher diversity of spacers than the others, suggesting more interactions present between AP1 and phages.

**Table 1:** Genomic overview of *Ca*. Acidulodesulfobacterales

	*Ca*. Acididesulfobacter		*Ca*. Acidulodesulfobacterium	
	AP1	AP2	AP3	AP4
Total length (bp)	2,459,539	2,217,123	1,873,120	2,093,156
No. of scaffolds	47	5	7	59
GC content (%)	29.88	32.69	37.35	35.52
Completeness (%)^a^	91.97	93.57	93.97	93.57
Contamination (%)^a^	0	0.80	0	0.80
No. of predicted genes	2208	1960	1805	2081
Hits to protein databases^b^				
NCBI-nr (%)	1817 (82.3)	1624 (82.9)	1553 (86.0)	1650 (79.3)
KEGG (%)	1791 (81.1)	1607 (82.0)	1551 (85.9)	1645 (79.0)
EggNOG (%)	1617 (73.2)	1458 (74.4)	1442 (77.9)	1500 (65.8)
16S rRNA	1	2	1	1
tRNA	55	52	54	44
No. of CRISPRs loci^c^	5	2	1	3

^a^ The completeness and contamination of the retrieved genomic bins were estimated by CheckM

^b^ Genes matching hits in different databases via BLASTx (*e*-value ≤ 10^−5^)

^c^ CRISPR loci of the retrieved genomes were annotated using CRISPRFinder

As mentioned above, relatively low OrthoANI values among these *Ca*. Acidulodesulfobacterales genomes might translate to differing metabolic potentials. Comparative analysis based on KEGG Orthology (KO) showed that 870 core KOs were shared by the four species, with 939 and 922 shared KOs in *Ca*. Acididesulfobacter (AP1 and AP2) and *Ca*. Acidulodesulfobacterium (AP3 and AP4), respectively (Supplementary Fig. S1 and Supplementary Table S3) (Materials and methods in the Supplementary Information). Notably, the number of species-specific KOs (59 in AP1, 21 in AP2, 41 in AP3, and 38 in AP4) was much lower than the core KOs, suggesting that genomic differences among these species were small. In spite of this, we found that for *Ca*. Acidulodesulfobacterales, urea metabolism was found only in AP1, and nitric oxide reduction occurred only in AP3. Metabolic comparisons among these species are also discussed in the subsequent section. These species-specific genes, and the functions they confer, likely enable these species to avoid competitive exclusion and thus coexist.

A total of 57 MAGs (with estimated completeness > 50 and <2% contamination) belonging to *Proteobacteria*, *Euryarchaeota*, *Nitrospirae*, *Micrarchaeota*, *Parvarchaeota*, *Firmicutes*, and *Actinobacteria* were also retrieved from the metagenomes of the artificial AMD system. The drastic change in relative abundances of these phyla over a 13-month period implied the potentially important roles of different dominant taxa at different times and that this ecosystem was in an unstable state (Supplementary Fig. S2). Subsequently, the relative abundances of the four species affiliated with *Ca*. Acidulodesulfobacterales and their transcripts were examined in the artificial AMD system over the 13-month period (Fig. 1b). The relative abundance of *Ca*. Acidulodesulfobacterales fluctuated between 4.7 and 61.5%. AP4 was the dominant species with relative abundance 4.2–42.0%, except in S5 when AP2 became the dominant species with relative abundance 9.4%. A similar trend was reflected in transcript abundance (Fig. 1b). The transcript abundance of *Ca*. Acidulodesulfobacterales ranged greatly from 7.5 to 70.6%, and AP4 had the highest transcript abundance (6.4−67.1%), except in S5, when AP2 showed the highest transcript abundance (~4.0%). There was a significant relationship (all *P* < 0.05) between the relative abundance of genomes (recruited from metagenomes) and transcript abundance for *Ca*. Acidulodesulfobacterales AP3, and AP4 (Supplementary Fig. S3), suggesting these lineages may not only be abundant, but also highly active.

### Metabolic potential of *Ca*. Acidulodesulfobacterales

**Fig. 3:**
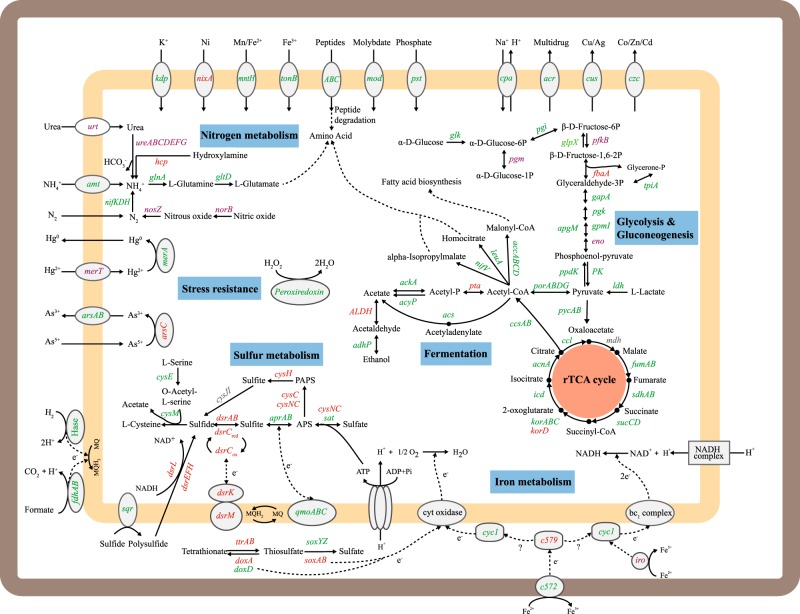
Metabolic capabilities of *Ca*. Acidulodesulfobacterales. Pathways associated with carbon, nitrogen, sulfur, and iron metabolism are shown, along with oxidative phosphorylation, fermentation, and stress response. Genes appearing in all four genomes, two or three genomes and one genome are shown in green, orange, and purple, respectively. Genes absent in all bins are gray. The copy numbers of each gene in the four bins are provided in Supplementary Table S4

**Fig. 4:**
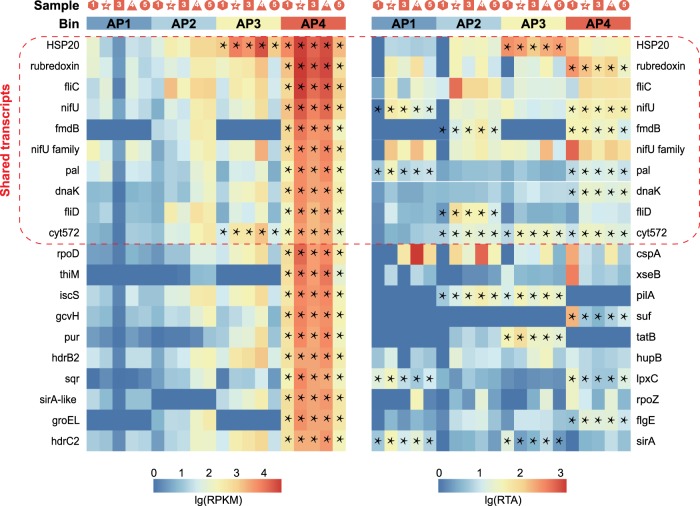
Expression profiles for 20 genes with the highest abundance (RPKM, left) and the highest relative transcriptional activity (RTA, right). Genes within the red dashed frame were shared by the two expression profiles. Asterisks indicate that a given transcript is significantly different in special bins comparing with in the other bins using one-way ANOVA analysis

Metabolic potentials of these species were constructed from the genomes (Fig. 3 and Supplementary Table S4), and further supported by expression of the genes, as evaluated through metatranscriptomics (Supplementary Tables S5 and S6). Overall, the 20 transcripts with the highest abundance (Reads Per Kilobase per Million mapped reads, RPKM) and the highest relative transcriptional activity (RTA) were characterized, among which 10 transcripts related to stress response (rubredoxin, *hsp20*, and *dnaK*), flagellar assembly (*fliC* and *fliD*), transport (pal), transcriptional regulation (*fmdB*), nitrogen fixation (*nifU* and *nifU* family maturases), and iron oxidation (*cyt572*) were shared by the two expression profiles of RPKM and RTA (Fig. 4). The unshared transcripts were associated with thiamine metabolism (*thiM* and *iscS*), methane metabolism (*hdrB2* and *hdrC2*), glyoxylate and dicarboxylate metabolism (*gcvH*), sulfur metabolism (*sqr*), sulfur relay system (*sirA* and *sirA*-like genes), metal-sulfur cluster assembly (*suf*), lipopolysaccharide biosynthesis (*lpxC*), pilus assembly (*pilA*), flagellar assembly (*flgE*), protein export (*tatB*), RNA degradation (*groEL*), and stress response (*rpoD*, *rpoZ*, *cspA*, *xseB,* and *hupB*) (Fig. 4). One-way analysis of variance (ANOVA) analysis with a Duncan test illustrated that RPKM levels for the top 20 transcripts of AP4 were always higher than the other species (all *P* < 0.05). But, at the RTA levels, this trend was not found. Supplementary Tables S5 and S6 provide additional details on key transcripts involved in carbon, nitrogen, sulfur, and iron metabolisms, oxidative phosphorylation and fermentation, and response to environmental stresses, which are individually described below.

#### Carbon fixation

As shown in Fig. 3, the reductive tricarboxylic acid (rTCA) cycle was identified in all four genomes, implying that *Ca*. Acidulodesulfobacterales have the genetic potential for carbon fixation via the rTCA cycle, in agreement with the fact that the rTCA cycle is often utilized by anaerobic or microaerobic species of *Proteobacteria*, especially *Deltaproteobacteria* [58]. Meanwhile, the transcriptional analysis confirmed the expression of genes in the rTCA cycle. Importantly, the key genes *ccsAB*, encoding citryl-CoA synthetase, were expressed at higher levels (RPKM) in AP4 than in the other three genomes (ANOVA, *P* = 0.004), suggesting *Ca*. Acidulodesulfobacterales (especially AP4) may be important autotrophs for the AMD community.

#### Nitrogen metabolism

The expression of the *nifDKH* genes, encoding nitrogenase, was confirmed in all of the four bins, illustrating that *Ca*. Acidulodesulfobacterales have the potential to fix nitrogen to synthesize organic nitrogen for growth. Among the four species, gene expression analysis revealed the higher expression level (RPKM) of nitrogenase in AP4 (ANOVA, *P* = 0.02), and no significant difference in the RPKM values of the *nifDKH* genes in *Ca*. Acidulodesulfobacterales across time. Also, the nitrogenase RTA in *Ca*. Acidulodesulfobacterales as a whole increased significantly (ANOVA, *P* = 0.004) across time, illustrating their increasing activity for nitrogen fixation. Meanwhile, we found the high expression of the genes *nifS* and *nifU* (as two of the top 20 transcripts), which are maturases required for the formation of the iron–sulfur cluster in nitrogenase [59], and the highest RPKM and RTA of the *nifU* gene were present in AP4. These findings provide evidence for an important role of *Ca*. Acidulodesulfobacterales, particularly AP4, in supplying nitrogen for the AMD community. Furthermore, statistical analyses demonstrated that RTA of the *nifDKH* genes in *Ca*. Acidulodesulfobacterales increased significantly (both *P* < 0.05) with the decrease of environmental pH and the increase of total iron (Supplementary Fig. S4a, b).

In addition to nitrogen fixation, *Ca*. Acidulodesulfobacterales could potentially reduce hydroxylamine to generate ammonium as evidenced by the expression of the *hcp* gene in AP1, AP2, and AP4. RTA analysis revealed the highest activity of the *hcp* gene in AP2. It is also noteworthy that AP1 harbored genes encoding urea transport system (*urtABCDE*) and urease (*ureDABCEFG*), indicating urea could potentially serve as an alternative nitrogen resource for AP1, similar to other known microorganisms (e.g., *Ferrovum* spp.) in AMD [60]. For ammonium transport and assimilation, the genes *amt*, *glnA*, and *gltD*, encoding ammonium transporter, glutamine synthetase, and glutamate synthase, respectively, were expressed in all four species, suggesting that *Ca*. Acidulodesulfobacterales likely gain organic nitrogen by ammonium assimilation for growth, like other microorganisms in AMD [60]. Interestingly, the *amt* gene showed the same trends in RPKM and RTA as the *nifDKH* genes, implying the enhanced demand and capacity of each member of the *Ca*. Acidulodesulfobacterales (especially AP4) to gain nitrogen for anabolism. Moreover, AP3 harbored a potential for reducing nitric oxide to nitrogen due to the detected expression of the *norB* and *nosZ* genes, which encode nitric oxide reductase subunit B and nitrous-oxide reductase, respectively. In summary, multiple strategies for the utilization of nitrogen resources (nitrogen, ammonium, urea etc.) were identified in *Ca*. Acidulodesulfobacterales, similar to *Leptospirillum* and a few other AMD taxa, as reported in previous studies [61–63].

#### Sulfur metabolism

**Fig. 5:**
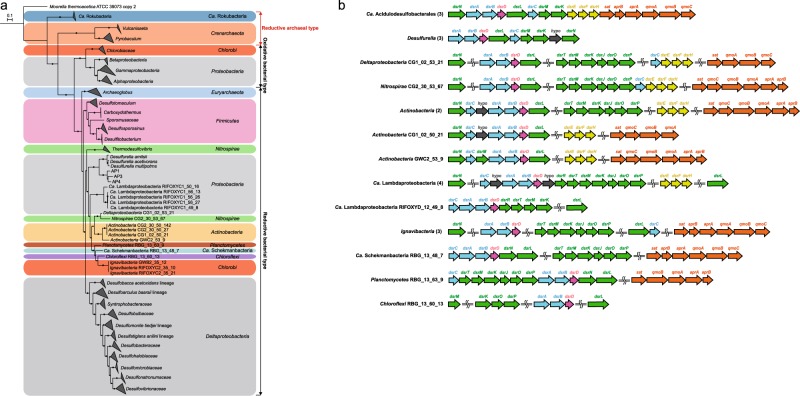
The dsr operon of *Ca*. Acidulodesulfobacterales. **a** Phylogenic analysis of the concatenated DsrAB proteins. Bootstrap values were based on 100 replicates, and only bootstrap values higher than 50% are shown with black circles. **b** The dsr operon structure in *Ca*. Acidulodesulfobacterales and previously reported genomes

One crucial characteristic of AMD is the high concentration of sulfate [7]. A complete dissimilatory sulfate reduction pathway was found in the genomes of AP1, AP3, and AP4, whereas the concatenated DsrAB protein tree demonstrated that the *dsrAB* genes from *Ca*. Acidulodesulfobacterales were the reductive type (Fig. 5a), showing that *Ca*. Acidulodesulfobacterales are potential acidophilic SRB (aSRB). The concatenated DsrAB protein tree also provides evidence for the fact that organisms of *Deltaproteobacteria* likely acquired the *dsrAB* genes in multiple lateral gene transfer events [64]. AP1, AP3, and AP4 were further confirmed as potential aSRB due to the *dsrD* gene present in their *dsr* operons, which was found widely in SRB but not in sulfur-oxidizing bacteria [65–67]. However, it is surprising to simultaneously detect the *dsrL* and especially *dsrEFH* genes (with well-known roles in sulfur oxidation) in the *dsr* operons of AP1, AP3, and AP4 (Fig. 5b). Previous studies have shown that the DsrL is a homolog of the small subunit of bacterial glutamate synthase and is essential for sulfur oxidation [68, 69]. The *dsrEFH* genes, which encode sulfur trafficking enzymes, are ubiquitous in sulfur oxidizers but absent in SRB [70, 71]. Given the occurrence of the *dsrL* and *dsrEFH* genes, these new species may be capable of oxidizing sulfide to sulfate via the reverse sulfate reduction pathway, analogous to the pathway model in *Desulfurivibrio alkaliphilus* [72]. To date, similar *dsr* operons have been found in several organisms from *Actinobacteria* (four genomes), *Ca*. Lambdaproteobacteria (four genomes), *Nitrospirae* (one genome), and *Deltaproteobacteria* (one genome), as shown in Fig. 5 [64]. Significant correlations (both *P* < 0.05) between the RTA values of the *dsrAB* and *dsrEFH* genes and between the RTA values of the *dsrAB* genes and the sulfate concentration were observed (Supplementary Fig. S4c, d). Considering the low concentration of oxygen in AMD bulk water (0.65–2.93 mg L^−1^), it is possible that planktonic *Ca*. Acidulodesulfobacterales or those on biofilm surfaces may be performing sulfide oxidation, whereas those within the biofilm matrix or anaerobic sediments may be able to simultaneously reduce sulfate. The latter process may be dominant in the environment, as evidenced by the much higher RTA of the *dsrD* gene than that of the *dsrEFH* genes. Therefore, we suggest that *Ca*. Acidulodesulfobacterales may not only reduce sulfate, but also oxidize sulfide, depending on oxygen concentrations and/or oxidation reduction potential in the environment. In addition, transcriptional results provided evidence that AP4 may play a more important role in sulfur metabolism than AP1 and AP3 due to its much higher expression level (RPKM) and RTA of the *dsrAB* genes (ANOVA, both *P* ≤ 0.05).

Interestingly, *Ca*. Acidulodesulfobacterales also harbored a partial pathway for assimilatory sulfate reduction due to their absence of the *cysJI* and *sir* genes, encoding sulfite reductase (Fig. 3). In addition, the *sqr* gene was expressed, with its highest RPKM and RTA in AP4 (ANOVA, both *P* < 0.05), suggesting that *Ca*. Acidulodesulfobacterales (especially AP4) have the potential to produce polysulfide, which plays an integral role in a wide variety of geochemical processes (e.g., pyrite formation, organic matter sulfidization, and isotope exchange among reduced sulfur species, and metal chelation) and thus contributes to detoxification and elemental burial and sequestration [73]. Furthermore, the presence of the *soxA*, *soxB*, *soxY*, and *soxZ* genes in AP1, AP2, and AP3 suggests that *Ca*. Acidulodesulfobacterales harbor the potential to oxidize S_2_O_3_^2-^ to produce S(0) via the partial SOX (sulfur oxidation) system without the *soxCD* genes [74]. The expression analysis of the *soxB* gene suggests that AP3 was highly active in oxidizing thiosulfate compared with the others (*t-*test, all *P* < 0.05). In addition, the *cysE* and *cysM* genes, encoding serine O-acetyltransferase and cysteine synthase B, respectively, were identified in all four genomes, illustrating that *Ca*. Acidulodesulfobacterales may utilize the generated sulfide to synthesize the l-cysteine and acetate for growth.

#### Iron metabolism

Considering the high availability of iron in AMD environments, genes involved in iron oxidation were searched for in the new genomes [60, 75]. Results revealed that all four genomes had *cyt572*- and *cyt1*-like genes, and only AP1 and AP4 had the *cyt579*-like gene (Supplementary Table S7). These cytochromes have been shown to participate in iron oxidation in *Leptospirillum* Group II and III [76], which are common in AMD environments [77]. As such, it is inferred that *Ca*. Acidulodesulfobacterales are likely able to oxidize iron and transfer electrons via the *cyt572*–*cyt579*–*cyc1* pathway. The key *cyt572*-like gene, one of the top 20 transcripts, showed a high expression level (RPKM) and a RTA in AP4. Moreover, an *iro*-like gene was identified in AP4 (Supplementary Table S7), which encodes the iron oxidase in *Acidithiobacillus ferrivorans* [75], demonstrating AP4 may be a potential Fe(II) oxidizer.

In addition to the above potential iron oxidation genes, genes encoding iron transport and storage proteins and the related regulators were also detected in *Ca*. Acidulodesulfobacterales. For Fe(II) transport, the double-copy gene *mntH* was confirmed in all four bins (Supplementary Table S5). Previous studies have demonstrated that the *mntH* gene encodes a secondary Fe(II) transporter with the primary function of Mn(II) uptake, as well as Fe(II) in certain circumstances [78]. Gene expression analysis uncovered that across time *Ca*. Acidulodesulfobacterales showed an increasing RTA of the *mntH* gene but no significant RPKM difference of the *mntH* gene was detected. AP4 was always the main contributor to the *mntH* RPKM, suggesting *Ca*. Acidulodesulfobacterales (especially AP4) as an important participant in iron metabolism. Also, four different TonB-dependent outer membrane Fe(III) siderophore transporter groups belonging to the CirA-linear and FepA-cyclic catecholate types were found in *Ca*. Acidulodesulfobacterales (Supplementary Table S8). These different types of siderophore receptors may provide a metabolic versatility for the growth of *Ca*. Acidulodesulfobacterales in AMD environments where iron co-precipitation with phosphates or sulfate during iron and sulfur biooxidation might compromise their ability to scavenge iron, as suggested for *Acidithiobacillus* spp. [78]. In most prokaryotes, diverse ferric uptake regulators (Fur) are responsible for iron regulation, and the iron storage proteins play a central role in maintaining iron homeostasis [78]. The present study revealed that only AP4 harbored the *fur* gene, but the *bfr* gene encoding bacterioferritin was expressed in all four bins with its highest RPKM in AP4. Ortholog analysis demonstrated that the bacterioferritin of *Ca*. Acidulodesulfobacterales was the classical iron storage protein due to the residue Met52, different from the Leu52 in the bacterioferritin of *Acidithiobacillus* spp. (Supplementary Fig. S5) [78]. Many studies have reported that the classical bacterioferritin can catalyze the oxidation and hydrolysis of iron at specific sites inside the protein shell, resulting in the formation of a mineral core of hydrated ferric oxide within the protein cavity, whereas it also has the capacity to re-utilize the iron stored inside its cavity and then release iron [79]. Thus, *Ca*. Acidulodesulfobacterales, particularly AP4, are likely to be important regulators of iron metabolism through complex iron management mechanisms.

#### Oxidative phosphorylation

Nearly complete pathways for oxidative phosphorylation along with high-affinity O_2_ terminal oxidases were found in the four bins (Fig. 4), indicating *Ca*. Acidulodesulfobacterales likely utilize oxygen as a terminal electron acceptor. All of the four bins harbored the *cydA* and *cydB* genes encoding cytochrome *bd* ubiquinol oxidase, which is a high-affinity terminal oxygen reductase capable of functioning under low oxygen concentrations [80, 81]. The RTA for *cydAB* genes was higher in AP4 and AP2 than in AP1 and AP3 (*t*-test, all *P* < 0.05), with the highest expression level (RPKM) in AP4. Furthermore, these species contained nearly complete NADH:quinone oxidoreductase (nuo) complexes (except the *nuoE*, *nuoF*, and *nuoG* genes) necessary for NADH-mediated oxidative phosphorylation and complete F-type ATPase, which is ubiquitous and phylogenetically conserved among *Bacteria* and considered to be the ancestral bacterial ATPase [82]. Thus, we propose that *Ca*. Acidulodesulfobacterales can respire oxygen in situ.

#### Fermentation

The present study revealed that *Ca*. Acidulodesulfobacterales are likely able to ferment, producing lactate (the *LDH* gene) and ethanol (the *adhP* and *ALDH* genes). Metatranscriptomic analysis revealed that among these four bins, AP4 had the lowest RTA of the *LDH* gene and the highest RTA of the *adhP* gene (ANOVA, all *P* < 0.05). The RPKM and RTA of the *adhP* gene was always higher than those of the *LDH* gene in AP4, implying that AP4 may prefer ethanol fermentation. AP1 and AP2 might perform acetate fermentation via the Pta-Ack pathway (the *pta*-*ackA* genes) that is widely distributed in bacteria [83], suggesting some *Ca*. Acidulodesulfobacterales are able to grow as acetogens. Thus, *Ca*. Acidulodesulfobacterales are likely to be facultative anaerobes.

#### Stress response

AMD is an extreme environment due to the low pH and high concentrations of dissolved metals [5, 84], where indigenous microbes must thus adapt to the extreme environmental stresses (including acid, heavy metals, and oxidation) for survival [60, 85]. For acid stress, the expression of the *kdpABC* and *kup* genes, encoding a complete potassium-transporting ATPase system and potassium uptake protein, respectively, indicated that *Ca*. Acidulodesulfobacterales may generate an inside-positive membrane potential by taking up K^+^ to partially deflect the inward flow of protons. Meanwhile, *Ca*. Acidulodesulfobacterales likely maintain a near-neutral cytoplasm by the metabolism of proton buffer molecules, such as phosphate (*pstSCAB*) and arginine (*pdaD*). These strategies respond to acid stress and have been widely used by AMD microbes [60, 85].

In response to heavy metal stress, diverse heavy metal transporters were expressed, such as the Cu(I)/Ag(I) efflux system (*cusAB*) and the divalent metal cation (Fe/Co/Zn/Cd) transporter (such as *copB* and *czcD*) (Supplementary Tables S5 and S6), suggesting that efflux of metal ions is an important strategy to resist against heavy metal stress for *Ca*. Acidulodesulfobacterales, as occurs in other known AMD microorganisms [60, 85]. Moreover, another strategy may be to reduce metal ions or metalloids (such as mercury) to less toxic reduced forms, which may then be exported.

For oxidative stress, genes encoding peroxiredoxin and thioredoxin reductase were expressed in *Ca*. Acidulodesulfobacterales, which are widely used to remit harm of oxidative stress by AMD microorganisms [86]. Besides, a novel cytoplasmic oxidative stress protection system composed of rubrerythrin (*rbr*) and rubredoxin (*rbo*, one of the top 20 transcripts) in anaerobic microbes [87] was found in *Ca*. Acidulodesulfobacterales, especially AP4, fully demonstrating that they were facing severe oxidative stress. Moreover, other proteins (such as cytochrome c551 peroxidase and thiol peroxidase) for the response of oxidative stress were also expressed in *Ca*. Acidulodesulfobacterales. In conclusion, all above findings explain the mechanisms how *Ca*. Acidulodesulfobacterales can survive in the extreme AMD environment.

## Concluding remarks

As abundant taxa in the extreme iron- and sulfur-bearing environments (including AMD ecosystems, Quaternary sediments, and deep-sea vents), *Ca. Acidulodesulfobacterales* was first detected through the 16S rRNA gene clone library 18 years ago [19]. We now describe the first four genomes affiliated with *Ca*. Acidulodesulfobacterales. This study demonstrated that ferrous iron was a main factor controlling the distribution pattern of *Ca*. Acidulodesulfobacterales in AMDs across Southeast China. The reconstruction of metabolic pathways with metatranscriptomics shed light on the metabolic versatility of *Ca*. Acidulodesulfobacterales, revealing that they are important participants in biogeochemical cycles of carbon, nitrogen, sulfur, and iron. Our work also provides insights into the mechanisms of adaptation and response to the extreme environmental stresses. As facultatively anaerobic autotrophs, *Ca*. Acidulodesulfobacterales likely use oxygen as terminal electron acceptor and thus couple Fe(II) and sulfide oxidation to aerobic respiration under aerobic conditions in AMD water and on biofilm surface, while they may perform dissimilatory sulfate reduction, Fe(III) respiration and fermentations under anaerobic conditions in the interior of biofilms and sediments. To further understand the physiological and genetic properties of *Ca*. Acidulodesulfobacterales, future studies should strive to obtain pure cultures or defined mixed cultures of these species to confirm these ecological functions. Meanwhile, future research should expand the number of genomes belonging to *Ca*. Acidulodesulfobacterales to further address questions related to biogeography, comparative genomics, and evolution. Such efforts will further expand our understanding of the taxonomic diversity and ecological role of the new order *Ca*. Acidulodesulfobacterales in nature.

## Supplementary information

Supplementary Information

Supplementary Table 1

Supplementary Table 2

Supplementary Table 3

Supplementary Table 4

Supplementary Table 5

Supplementary Table 6

Supplementary Table 7

Supplementary Table 8
