# Supplementary material for: Insights into ecological role of a new deltaproteobacterial order Candidatus Acidulodesulfobacterales by metagenomics and metatranscriptomics
Source: ISME J. 2019 Apr 8;13(8):2044–57. doi: 10.1038/s41396-019-0415-y (PMC6776010; doi:10.1038/s41396-019-0415-y)
Supplement: Supplementary file 1 — Supplementary Information [file 41396_2019_415_MOESM1_ESM.docx]

Supplementary Information

**Insights into ecological role of a new** **deltaproteobacterial order** ***Candidatus* Acidulodesulfobacterales** **by** **metagenomics and** **metatranscriptomics**

Running title: Ecological role of *Ca.* Acidulodesulfobacterales

Sha Tan^1,^*, Jun Liu^1,2,^*, Yun Fang^3,^*, Brian P. Hedlund^4,5^, Zheng-han Lian^1,6^, Li-ying Huang^1^, Jin-tian Li^7^, Li-nan Huang^1^, Wen-jun Li^1^, Hong-chen Jiang^3^, Hai-liang Dong^8,2,#^, and Wen-sheng Shu^7,#^

^1^State Key Laboratory of Biocontrol, Guangdong Key Laboratory of Plant Resources, School of Life Sciences, Sun Yat-sen University, Guangzhou 510275, PR China.

^2^Department of Geology and Environmental Earth Science, Miami University, OH 45056, USA.

^3^State Key Laboratory of Biogeology and Environmental Geology, China University of Geosciences, Wuhan 430074, PR China.

^4^School of Life Sciences, University of Nevada, Las Vegas, Las Vegas, NV 89154, USA.

^5^Nevada Institute of Personalized Medicine, University of Nevada, Las Vegas, Las Vegas, NV 89154, USA.

^6^Guangdong Magigene Biotechnology Co. Ltd., Guangzhou 510000, PR China.

^7^School of Life Sciences, South China Normal University, Guangzhou 510631, PR China.

^8^State Key Laboratory of Biogeology and Environmental Geology, China University of Geosciences, Beijing 100083, PR China.

*These authors contributed equally to this work.

^#^Corresponding authors:

Wen-sheng Shu, zsushuwensheng@163.com, School of Life Sciences, South China Normal University, Guangzhou 510631, PR China.

Hai-liang Dong, dongh@miamioh.edu, Department of Geology and Environmental Earth Science, Miami University, Oxford, OH 45056, USA.

**Materials and methods**

**Physicochemical analyses**

Physicochemical characteristics were measured as previously reported [1], and summarized in Table S1. Briefly, solution pH, dissolved oxygen (DO), electrical conductivity (EC) and redox potential (Eh) were measured onsite using specific electrodes. Total organic carbon (TOC; TOC-VCPH; Shimadzu, Kyoto, Japan) and total nitrogen (TN; SmartChem200; WESTCO Scientific Instruments Inc., Brookfield, CT) were determined according to standard methods [2]. The concentrations of ferrous iron (Fe^2+^) and ferric iron (Fe^3+^) were determined with the 1, 10-phenanthroline method at 530 nm [3], and the concentration of sulfate was measured with the BaSO_4_-based turbidimetric method [4]. Some heavy metals (including Mn, Zn, Cr, Cu, As, Cd, and Pb) were measured with inductively coupled plasma optical emission spectrometry (ICP-OES; Optima 2100DV, PerkinElmer, Massachusetts, USA) after the collected acidic water samples were digested at 180 ℃ with a HNO_3_/HCl mixture (1:3, v v^-1^).

**DNA and RNA extraction, and metagenomic and metatranscriptomic sequencing**

Genomic DNA was extracted from the cell pellets as described previously [5]. Approximately 5 µg of DNA were fragmented using a Covaris M220 ultrasonicator (Covaris, Woburn, MA, USA), and DNA integrity and fragment size were determined on an Agilent 2100 Bioanalyzer (Agilent, Santa Clara, CA, USA). Standard shotgun libraries of 300 bp in insert size were constructed using the NEBNext Ultra DNA Library Prep Kit, and visualized with an Agilent 2100 Bioanalyzer for the quality. Subsequently qualified libraries were sequenced using an Illumina MiSeq platform (paired-end 300-bp mode), an Illumina HiSeq 2500 platform (paired-end 250-bp mode) or an Illumina HiSeq 4000 platform (paired-end 150-bp mode) at the Novogene Company (Beijing, China). Total cellular RNA was extracted using the RNeasy Mini kit (Qiagen) according to the manufacturer’s instructions. Total RNA was transported to the Novogene Company (Beijing, China) on dry ice for subsequent rRNA subtraction, RNA amplification, cDNA synthesis, library construction, and sequencing with an Illumina HiSeq 4000 platform (paired-end 150-bp mode).

**KEGG Orthology based cluster analyses of** ***Ca.* Acidulodesulfobacterales genomes**

Comparative genomic analysis was performed based on KEGG Orthology as described in a previous study [6]. The occurrence of KEGG Orthology (KO) in each *Ca.* Acidulodesulfobacterales genome was summarized based on KEGG annotation results (Table S3), and then the data was transformed to a (0, 1) format. Clustering analysis of all genomes affiliated with *Ca.* Acidulodesulfobacterales was performed based on the (0, 1) data format, visualized with Venn diagrams.

**Calculation of relative abundance and transcript abundance**

The coverage of each scaffold in each sample was calculated by separately mapping the high-quality reads from each metagenomic dataset to these manually curated co-assemblies with BBMap as described above. Then as previously reported [7], the coverage of each bin was calculated as the average of scaffold coverages, weighting each scaffold by its length in base pairs, and then the relative abundance of each bin in each sample was calculated as its coverage divided by the total coverage of all genomes in each metagenomic dataset.

Raw metatranscriptomic reads were pre-processed in the same manner as for metagenomic reads. The rRNA sequences (5S, 16S and 23S) were removed by SortMeRNA (version 2.180) [8]. Subsequently these high-quality transcriptomic reads were mapped to the predicted protein-coding genes from the metagenomic co-assembly using BBMap, and the expression level for each gene was normalized to reads per kilobase per million mapped reads (RPKM). The present contribution of each bin was calculated as the ratio of the RPKM sum of all transcripts within a bin to the RPKM sum of the total metatranscriptome as previously described [9]. In addition, relative transcriptional activity (RTA) of genes from the four retrieved genomes in each sample was determined by dividing the normalized cDNA abundance by the normalized DNA abundance of the same gene [10]. The normalized cDNA abundance was calculated as the ratio of the RPKM of each gene to the RPKM sum of the total genes in the genome.

**Results and discussion**

**The taxonomic epithets for AP1-4**

Based on the data presented here, we propose the taxonomic epithets “*Ca.* Acidulodesulfobacterium ferriphilum” (AP3), “*Ca.* Acidulodesulfobacterium acidiphilum” (AP4), “*Ca.* Acididesulfobacter guangdongensis” (AP2), and “*Ca.* Acididesulfobacter diazotrophicus” (AP1). The etymology and descriptions are as follows: “Acidulodesulfobacterium” (A.cid.u.lo.de.sul.fo.bac.te’ri.um) L. adj. *acidula*, tart, acidic; L. pref. *de*, from; L. n. *sulfur*, sulfur; N.L. pref. desulfo- (a dissimilatory sulfate-reducing bacterium); L. neut. n. bacterium, a rod; N.L. neut. n. *Acidulodesulfobacterium*, a rod-shaped sulfate reducer in acidic environments. The type species is “*Ca.* Acidulodesulfobacterium ferriphilum”. “*Ca.* Acidulodesulfobacterium ferriphilum” (fer.ri’phi.lum) L. n. ferrum, iron; N.L. neut. adj. philum Gr. adj. philos loving; M.L. neut. adj. ferriphilum, iron-loving. The type material is the metagenomic bin AP3. “Ca. Acidulodesulfobacterium acidiphilum” (a.ci.di’phi.lum. M.L. neut. n. acidum an acid; Gr. adj. philos loving; M.L. neut. adj. acidiphilum acid-loving). The type material is the metagenomic bin AP4. “Acididesulfobacter” (a.ci.di.de.sul.fo.bac’ter) L. masc. adj. acidus acid; L. pref. *de*, from; L. n. *sulfur*, sulfur; N.L. pref. desulfo- (a dissimilatory sulfate-reducing bacterium); L. neut. n. bacter, a rod; N.L. neut. n. *Acididesulfobacter*, a rod-shaped sulfate reducer in acidic environments. The type species is “*Ca.* Acididesulfobacter guangdongensis”. “*Ca.* Acididesulfobacter guangdongensis” (guang.dong.en’sis) N.L. masc. n. guangdongensis, pertaining to Guangdong, a province of southern China, where this organism was first genomically described. The type material is the metagenomic bin AP2. “*Ca.* Acidulodesulfobacterium diazotrophicus” (di.a.zo.tro’phi.cus) L. inseparable particle dis, twice, doubly; N.L. n. azotum [from Fr. n. azote (from Gr. prep. a, not; Gr. n. zôê, life; N.Gr. n. azôê, not sustaining life)], nitrogen; N.L. pref. diazo-, pertaining to dinitrogen; N.L. adj. trophicus (from Gr. adj. trophikos), nursing, tending; N.L. masc. adj. diazotrophicus, growing on dinitrogen. The type material is the metagenomic bin AP1. In addition, phylogenomic analyses support the proposal for a candidate family inclusive of both “*Ca.* Acidulodesulfobacterium” and “*Ca.* Acididesulfobacter”, “Acidulodesulfobacteraceae” (A.cid.u.lo.de.sul.fo.bac.ter.a’ce.ae) N.L. n. Acidulodesulfobacterium type genus of the family; L. suff. -aceae ending to denote a family; N.L. fem. pl. n. Acidulodesufobacteraceae the family of the genus Acidulodesulfobacterium. Similarly, a candidate order is proposed, “Acidulodesulfobacterales” (A.cid.u.lo.de.sul.fo.bac.ter.a’les) N.L. n. Acidulodesulfobacterium type genus of the order; L. suff. -ales ending to denote a family; N.L. fem. pl. n. Acidulodesufobacterales the family of the genus Acidulodesulfobacterium.

**References**

1. Kuang JL, Huang LN, Chen LX, Hua ZS, Li SJ, Hu M, et al. Contemporary environmental variation determines microbial diversity patterns in acid mine drainage. *ISME J*. 2013; **7**: 1038-1050.
2. Page AL, Miller RH, Keeney DR, Baker D, Ellis R, Rhoades J (eds). *Methods of Soil Analysis Part 2: Chemical and Microbiological Properties*, 2rd end. ASA/SSSA Publisher Inc: Madison, WI, USA, 1982.
3. Hill AG BE, Coles LE, McLaughlan EJ, Meddle DW, Pater MJ et al. Standardised general method for the determination of iron with 1,10-phenanthroline. *Analyst*. 1978; **103**: 391-396.
4. Chesnin L, Yien CH. Turbidimetric determination of available sulfates. *Soil Sci Soc Am J*. 1951; **15**: 149-151.
5. Fang Y, Xu M, Chen X, Sun G, Guo J, Wu W, et al. Modified pretreatment method for total microbial DNA extraction from contaminated river sediment. *Front Environ Sci Eng*. 2015; **9**: 444-452.
6. Chen LX, Méndez-García C, Dombrowski N, Servín-Garcidueñas LE, Eloe-Fadrosh EA, Fang BZ, et al. Metabolic versatility of small archaea *Micrarchaeota* and *Parvarchaeota*. *ISME J*. 2017; **12**: 756-775.
7. Woodcroft BJ, Singleton CM, Boyd JA, Evans PN, Emerson JB, Zayed AA, et al. Genome-centric view of carbon processing in thawing permafrost. *Nature*. 2018; **560**: 49-72.
8. Kopylova E, Noé L, Touzet H. SortMeRNA: fast and accurate filtering of ribosomal RNAs in metatranscriptomic data. *Bioinformatics*. 2012; **28**: 3211-3217.
9. Jewell TNM, Ulas K, Brodie EL, Williams KH, Beller HR. Metatranscriptomic evidence of pervasive and diverse chemolithoautotrophy relevant to C, S, N and Fe cycling in a shallow alluvial aquifer. *ISME J*. 2016; **10**: 2106-2117.
10. Hua ZS, Han YJ, Chen LX, Liu J, Hu M, Li SJ, et al. Ecological roles of dominant and rare prokaryotes in acid mine drainage revealed by metagenomics and metatranscriptomics. *ISME J*. 2015; **9**: 1280-1294.

**Figures**

**
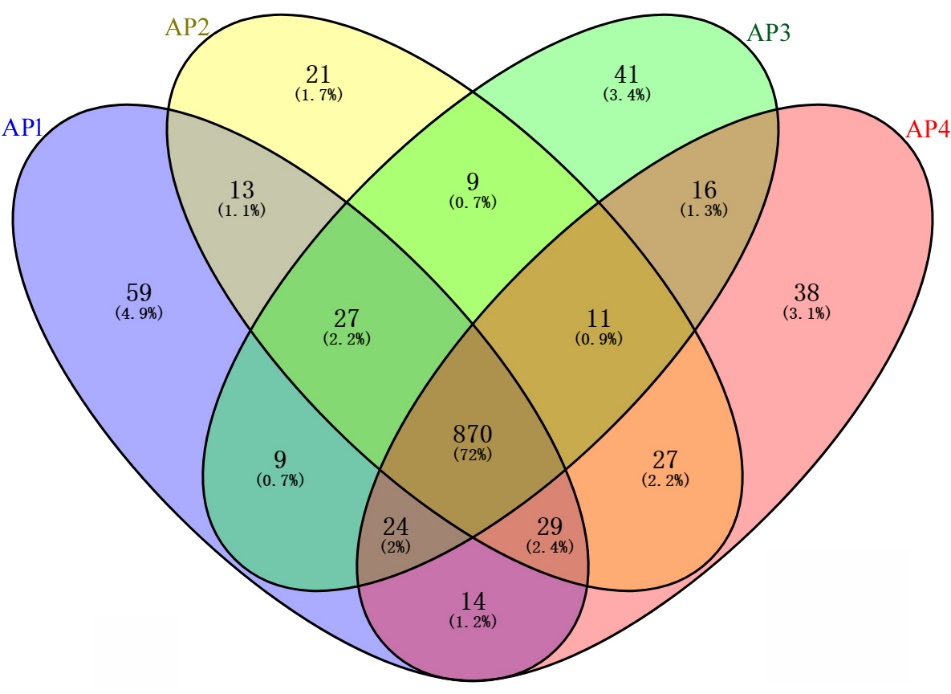
**

**Fig. S1** Venn diagram depicting the shared and unique KOs in the genomes of *Ca.* Acidulodesulfobacterales. The numbers shown in the Venn diagram indicate the number of the shared or species-specific KOs.

**
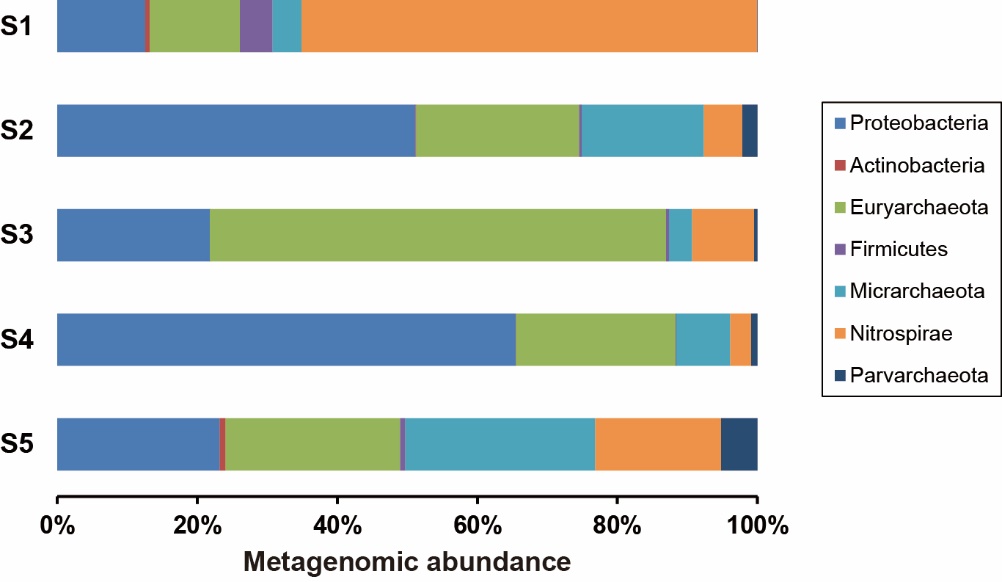
**

**Fig. S2** Metagenomic abundance of phyla across time. S1 through S5 represent July 2016, August 2016, December 2016, February 2017, and August 2017 samples, respectively.

**
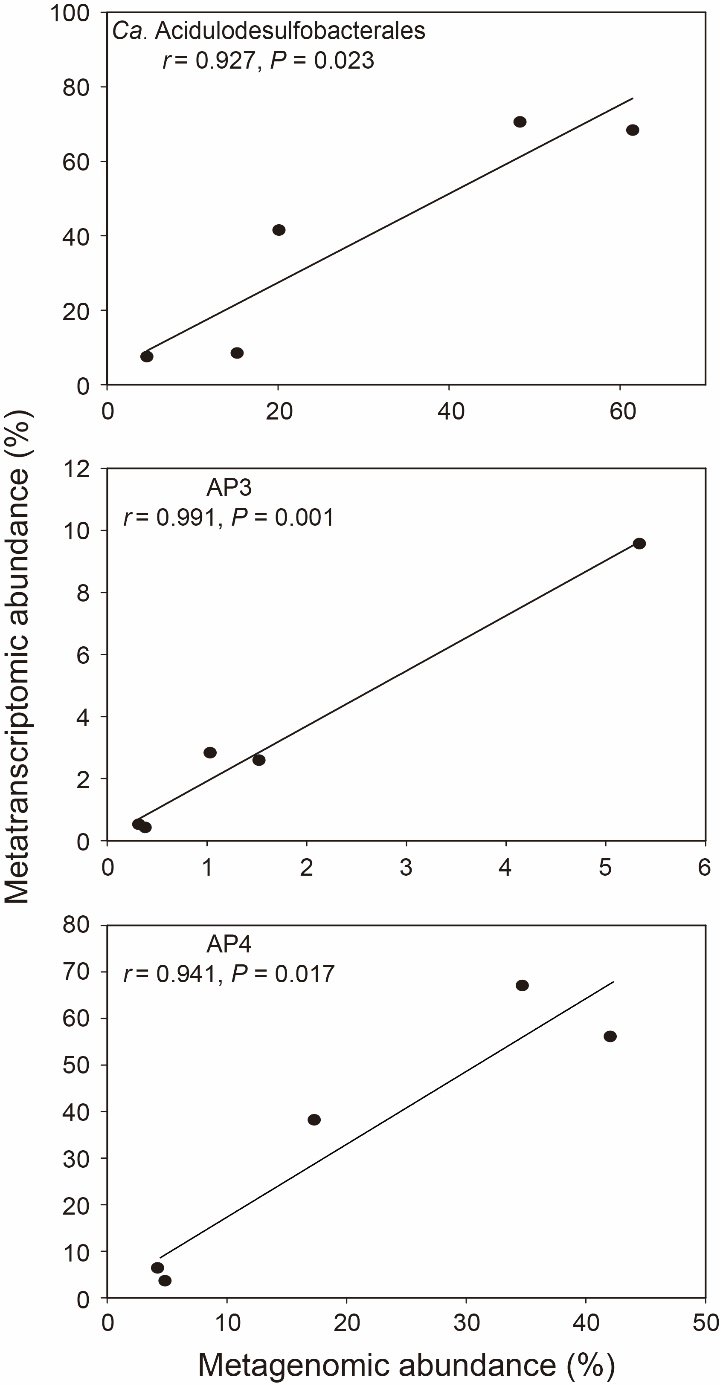
**

**Fig. S3** Correlations between metagenomic abundance and metatranscriptomic abundance for *Ca.* Acidulodesulfobacterales, AP3 and AP4 using a linear model. Pearson correlation coefficients (*r*) are shown with the associated Bonferroni-corrected *P* values.


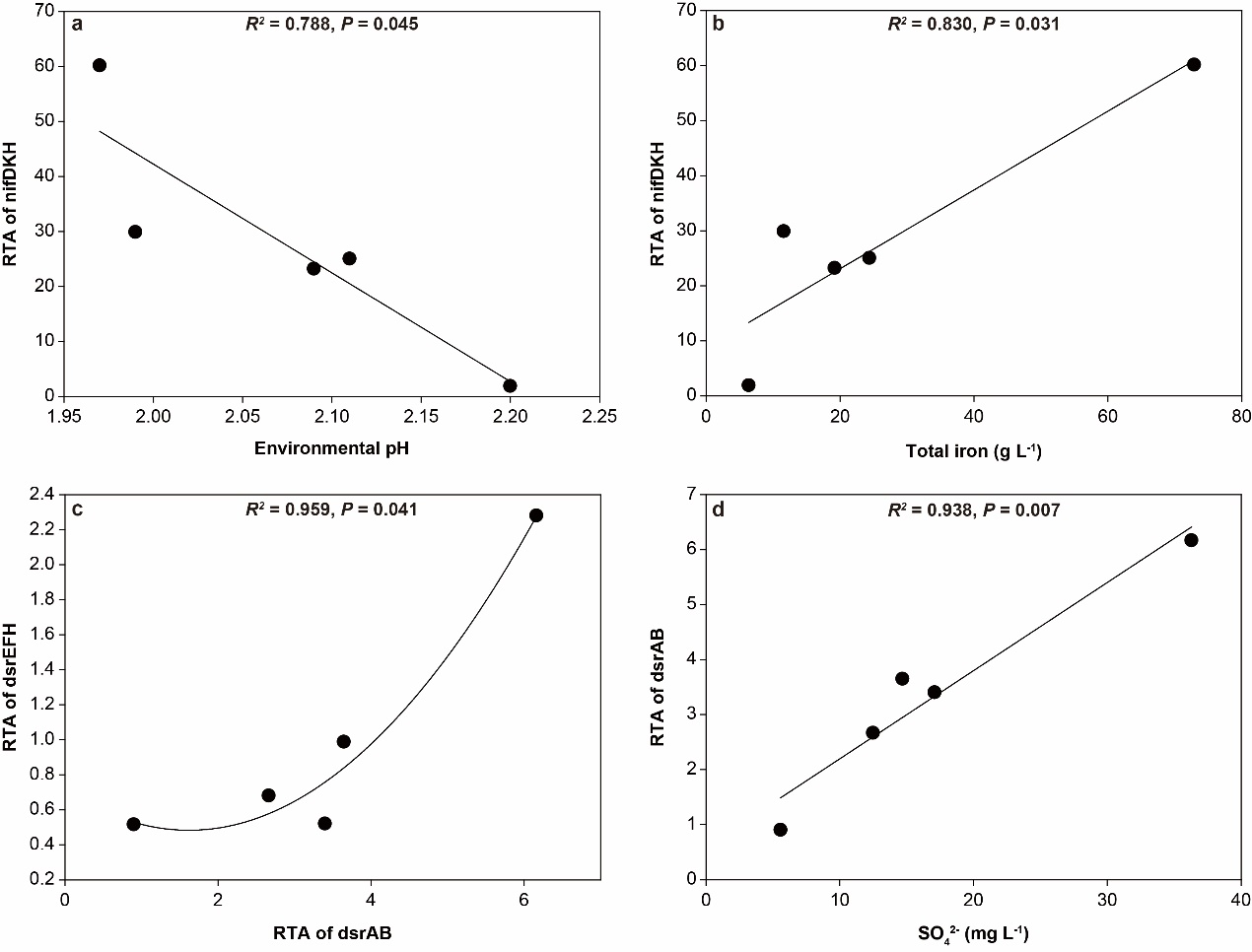


**Fig. S4** Correlations between RTA of *nifDKH* and pH and total iron, and RTA of *dsrAB* and RTA of *dsrEFH* and sulfate concentration.


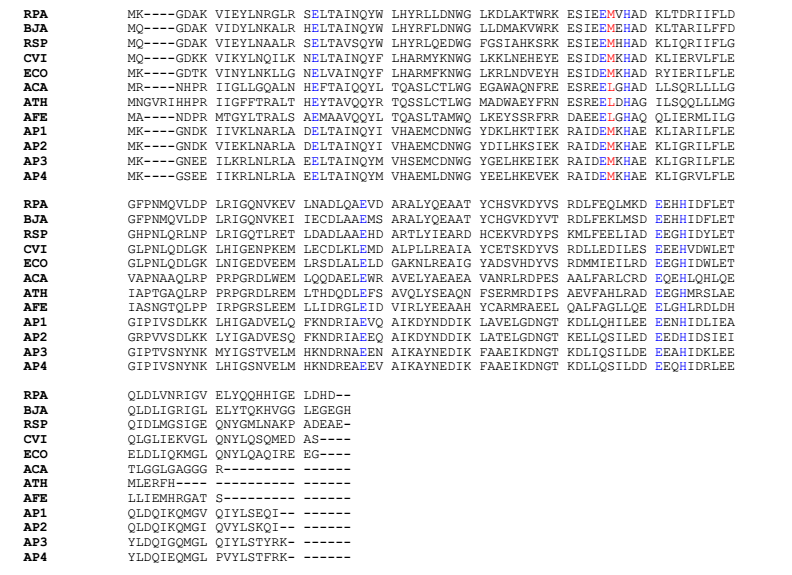


**Fig. S5** Sequence alignment of bacterioferritins.

Abbreviations: RPA, *Rhodopseudomonas palustris*; BJA, *Bradyrhizobium japonicum*; RSP, *Rhodobacter sphareoides*; CVI, *Chromobacterium violaceum*; ECO, *E. coli*; ACA, *Acidithiobacillus caldus*; ATH, *Acidithiobacillus thiooxidans*; AFE, *Acidithiobacillus ferrooxidans*. The binuclear metallic center is indicated in blue and the heme ligand in red.
