# Supplementary material for: Insights into ecological role of a new deltaproteobacterial order Candidatus Acidulodesulfobacterales by metagenomics and metatranscriptomics
Source: ISME J. 2019 Apr 8;13(8):2044–57. doi: 10.1038/s41396-019-0415-y (PMC6776010; doi:10.1038/s41396-019-0415-y)
Supplement: Supplementary file 2 — Supplementary Table 1 [file 41396_2019_415_MOESM2_ESM.docx]

**Table S1** Physicochemical properties of five samples collected from an artificial pyrite pond

|  | **pH** | **DO** | **EC** | **Eh** | **TC** | **TN** | **TFe** | **Fe^2+^** | **SO_4_^2-^** | **Mn** | **Zn** | **Cr** | **Cu** | **As** | **Cd** | **Pb** |
| --- | --- | --- | --- | --- | --- | --- | --- | --- | --- | --- | --- | --- | --- | --- | --- | --- |
| **S1** | 2.20 | 2.46 | 19.8 | 438 | 85.9 | 60.9 | 6.38 | 0.78 | 5.59 | 1157 | 412 | 1.17 | 1.47 | 2.79 | 0.98 | 1.06 |
| **S2** | 2.09 | 2.93 | 42.5 | 374 | 114 | 63.6 | 19.2 | 6.93 | 12.5 | 2639 | 1071 | 1.45 | 0.69 | 1.24 | 2.40 | 0.32 |
| **S3** | 2.11 | 0.65 | 47.4 | 344 | 119 | 85.2 | 24.4 | 9.49 | 17.1 | 3942 | 1528 | 1.26 | 0.54 | 1.51 | 3.19 | 0.21 |
| **S4** | 1.97 | 1.06 | 45.4 | 381 | 123 | 39.0 | 72.9 | 1.73 | 14.7 | 3294 | 1307 | 1.52 | 0.84 | 1.57 | 2.87 | 0.20 |
| **S5** | 1.99 | 2.17 | 17.7 | 433 | 45.5 | 47.6 | 11.6 | 7.81 | 36.3 | 1373 | 832 | 8.20 | 2.37 | 53.1 | 1.53 | 0.86 |

DO, dissolved oxygen (mg ml^-1^); EC, electrical conductive (ms cm^−1^); Eh, redox potential (mV); TC, total carbon (mg L^-1^); TN, total nitrogen (mg L^-1^); TFe, total Fe (g L^-1^); Fe^2+^ (g L^-1^); SO_4_^2-^ (g L^-1^); Mn (mg L^-1^); Zn (mg L^-1^); Cr (mg L^-1^); Cu (mg L^-1^); As (mg L^-1^); Cd (mg L^-1^); Pb (mg L^-1^).

S1 through S5 represent July 2016, August 2016, December 2016, February 2017, and August 2017 samples, respectively.
